# Effect of lemborexant on sleep-wake state discrepancy in participants with insomnia disorder

**DOI:** 10.1007/s00213-025-06845-4

**Published:** 2025-06-24

**Authors:** Kent Naito, Kunihiro Iwamoto, Seiko Miyata, Ippei Okada, Keita Kawai, Akihiro Fujimoto, Yuki Kogo, Daisuke Mori, Nao Matsuyama, Masahiko Ando, Norio Ozaki, Masashi Ikeda

**Affiliations:** 1 Department of Psychiatry, Nagoya University Graduate School of Medicine, 65 Tsurumai-cho, Showa-ku, Nagoya, 466-8550 Aichi Japan; 2 Medical Headquarters, Eisai Co., Ltd., Tokyo, Japan; 3 Brain and Mind Research Center, Nagoya University, Nagoya, Japan; 4 Pathophysiology of Mental Disorders, Nagoya University, Graduate School of Medicine, Nagoya, Japan; 5 Ascent Development Services, Tokyo, Japan; 6 Department of Advanced Medicine, Nagoya University Hospital, Nagoya, Japan

**Keywords:** Insomnia disorder, Sleep-wake state discrepancy, Objective sleep, Subjective sleep, Lemborexant, Daytime functioning

## Abstract

**Rationale:**

The efficacy of hypnotic drugs for sleep-wake state discrepancy remains unclear.

**Objective:**

This study evaluated the efficacy of lemborexant in improving sleep-wake state discrepancy in insomnia disorder and its associations with sleep quality and daytime functioning.

**Methods:**

Twenty-nine participants diagnosed with insomnia disorder were treated with lemborexant for 12 weeks. Objective sleep parameters were measured using a home electroencephalography device, and subjective parameters were obtained from electronic sleep diaries. Sleep-wake state discrepancy indices, including discrepancies of sleep onset latency (disSOL), wake after sleep onset (disWASO), total sleep time (disTST), and sleep efficiency (disSE), were calculated as objective minus subjective sleep parameters. The Pittsburgh Sleep Quality Index (PSQI) and Epworth Sleepiness Scale (ESS) were used to assess sleep quality and daytime sleepiness. Linear mixed-effects models were used for data analysis.

**Results:**

Both subjective and objective sleep parameters were improved at 12 weeks after lemborexant administration. The disTST decreased significantly at 4 and 12 weeks. The disSOL increased and disSE decreased significantly by 12 weeks. Participants with defined sleep-wake state discrepancy (disTST > 0 min) showed greater decreases in these indices, whereas minimal changes were observed in those without. The disWASO did not change significantly among baseline and 4 and 12 weeks. The improvement in disTST demonstrated a significant association with the reduction of ESS scores (*p* = 0.008), whereas there was no substantial effect on PSQI scores.

**Conclusions:**

The present findings suggest that lemborexant treatment reduces sleep-wake state discrepancy, which may be associated with improved daytime functioning in patients with insomnia disorder.

## Introduction

Insomnia, one of the most prevalent sleep disturbances, affects approximately 15–20% of the adult population worldwide, with insomnia disorder involving chronic, persistent insomnia impacting up to 10% of individuals (Morin and Buysse 2024). Insomnia is associated with adverse long-term outcomes, including increased risks of depression, anxiety, cardiovascular disease, and work disability (Morin and Buysse 2024). Though the evaluation and management of insomnia disorder predominantly rely on subjective reports in real-world clinical practice, including sleep diaries and questionnaires, a significant discrepancy between subjective and objective sleep measures, termed sleep-wake state discrepancy, is frequently observed (Stephan and Siclari 2023). This phenomenon reflects the complex interplay between subjective experience and underlying physiological factors in insomnia disorder (Stephan and Siclari 2023).

Sleep-wake state discrepancy, reported in 9.2–50% of patients with insomnia, is also recognized in other sleep disorders, including obstructive sleep apnea, and it has been associated with psychological conditions such as depression and anxiety, highlighting its multifaceted etiology (Rezaie et al. 2018). This sleep-wake state discrepancy presents several significant clinical challenges (Castelnovo et al. 2019; Rezaie et al. 2018; Stephan and Siclari 2023). First, clinical management is complex because it requires both objective and subjective assessments, and the lack of treatment guidelines makes it difficult to determine appropriate therapeutic approaches, potentially leading to unnecessary medication use if the patient does not perceive improvement. Sleep-wake state discrepancy is also associated with psychological distress and anxiety, which can exacerbate comorbid psychiatric disorders and result in significant impairment of daily activities and increased daytime fatigue (Rezaie et al. 2018; Wenigmann et al. 2019).

Cognitive-behavioral therapy for insomnia (CBT-I) is considered the first-line intervention for insomnia disorder, and it has demonstrated efficacy in treating sleep-wake state discrepancy (Dzierzewski et al. 2019; Perrault et al. 2022; Spina et al. 2024). Pharmacotherapy is recommended when CBT-I is ineffective or inaccessible (Edinger et al. 2021; Riemann et al. 2023). However, the efficacy of pharmacological interventions for sleep-wake state discrepancy remains largely unexplored, representing a significant gap in our understanding of treatment options (Rezaie et al. 2018; Stephan and Siclari 2023).

The evaluation of sleep-wake state discrepancy requires comprehensive assessment integrating both subjective and objective measures. Whereas polysomnography (PSG) and actigraphy are frequently used for objective sleep assessment, both methods have limitations. PSG’s artificial setting may not reflect natural sleep patterns, and discrepancies exist between actigraphy and sleep diary data. Recent studies have shown that a portable electroencephalography (EEG) device offers advantages over PSG, being less restrictive and allowing repeated measurements in the usual sleep environment. Moreover, it provides data more consistent with subjective sleep reports (Kawai et al. 2023; Miyata et al. 2021).

The FLUID study, a 12-week, prospective investigation of lemborexant’s effects in participants with insomnia disorder, used home-based assessments combining sleep diaries with a wearable EEG device (Miyata et al. 2024; Okada et al. 2021). This approach enabled ecological assessment of sleep patterns, demonstrating significant improvements in both subjective and objective sleep parameters. In addition, the study showed that lemborexant treatment significantly improved sleep quality and daytime sleepiness. The present study represents a secondary analysis of the FLUID study data, specifically evaluating lemborexant’s efficacy in treating sleep-wake state discrepancy in middle-aged and older participants with insomnia disorder. By using home-based assessment and rigorous evaluation methods, this study aimed to address critical gaps in our understanding of pharmacotherapy for sleep-wake state discrepancy while elucidating the clinical significance of changes in sleep-wake state discrepancy and their therapeutic implications.

## Methods

### Participants

This study included 29 patients (mean age: 58.5 ± 7.6 years; male/female: 15/14) diagnosed with insomnia disorder based on the Diagnostic and Statistical Manual of Mental Disorders, Fifth Edition. There were 31 participants in the FLUID study, but 29 participants with no missing values for sleep-wake state discrepancy before medication were selected for this study. Patients with moderate-to-severe obstructive sleep apnea (3% oxygen desaturation index (3%ODI) > 40/h) and periodic limb movement disorder were excluded using pulse oximetry (PULSOX-Me300; Teijin Pharma, Tokyo, Japan) and portable electromyography (EMG logger; GC, Tokyo, Japan). No comorbid psychiatric or neurological disorders were identified during physicians’ interviews. The concurrent use of hypnotics was prohibited, whereas the use of internal medicine drugs and over-the-counter medications was permitted unless otherwise listed in the protocol article. Details of inclusion and exclusion criteria of study participants are available in previous reports (Miyata et al. 2024; Okada et al. 2021).

### Procedures

Participants were included through a screening phase and underwent objective and subjective sleep assessments before (baseline) and 4 and 12 weeks after starting medication. Participants received a starting dose of 5 mg of lemborexant after the baseline assessment and were allowed to increase the dose to 10 mg if ineffective after 4 weeks.

### Assessments

#### Objective sleep assessment

Objective sleep parameters were assessed by home sleep testing using the Zmachine Insight+ (General Sleep, Cleveland, OH, USA), a single-channel EEG that monitors sleep and provides algorithm-based sleep staging (Miyata et al. 2021; Wang et al. 2015). The following four parameters were evaluated from one night’s data at baseline and after 4 and 12 weeks of treatment: objective sleep onset latency (oSOL; time elapsed to the onset of a 12-minute period, of which 10 minute were recorded as sleep); objective wake after sleep onset (oWASO; total minutes awake after oSOL); objective total sleep time (oTST; accumulation of all epochs determined to represent sleep during the time in bed); and objective sleep efficiency (oSE; proportion of time spent asleep per total recording time; TRT, calculated as TST/TRT×100) scores.

#### Subjective sleep assessment

Subjective sleep parameters were derived from electronic sleep diaries, and subjective SOL (sSOL), subjective WASO (sWASO), subjective TST (sTST), and subjective SE (sSE) scores were evaluated. Each sleep diary parameter was calculated from baseline and one-night data from weeks 4 and 12 of treatment.

#### Sleep-wake state discrepancy evaluation

Sleep-wake state discrepancy was assessed using differences between objective measures obtained with the Zmachine Insight + and subjective sleep measures recorded in sleep diaries. Sleep-wake state discrepancy was calculated as the most common difference (= objective measure– subjective measure), and the defined sleep-wake state discrepancy was a TST deviation at baseline (oTST – sTST > 0). The TST absolute discrepancy has been the most widely used measure in previous studies (Stephan and Siclari 2023). It is preferable to define a sleep-wake state discrepancy by TST misperception rather than SOL misperception, and a comprehensive interpretation is necessary, because sleep-wake state discrepancy is not limited to TST, but also extends to SOL, WASO, and SE (Castelnovo et al. 2019). The primary outcomes were the change in sleep-wake state discrepancy (disSOL, disWASO, disTST, and disSE) from baseline to weeks 4 and 12, respectively. An increase in disSOL and disWASO indicated improvement, whereas a decrease in disTST and disSE indicated improvement. Baseline measurements were missing for two participants, so data from the screening period were used as a proxy.

### Questionnaires

Participants completed the questionnaires at each assessment point, including the Pittsburgh Sleep Quality Index (PSQI) (Buysse et al. 1989) and Epworth Sleepiness Scale (ESS) (Johns 1991). The Beck Depression Inventory-II (BDI-II) (Beck et al. 1996) was also assessed as a factor that may influence sleep-wake state discrepancy (Kawai et al. 2022).

### Statistical analysis

Statistical analyses were conducted using R software (version 4.4.1; R Foundation for Statistical Computing, Vienna, Austria; https://www.r-project.org/). There were missing values in 9 cases at week 4 and in 12 cases at week 12. Since these missing values were missing completely at random (Little MCAR test, *p* > 0.05), 50 multiply imputed datasets were created by multiple imputation (mice package). The data were analyzed using linear mixed-effects models (lme4 package) chosen for their ability to account for dependency in the data due to the repeated measures. Models were fitted to sleep-wake state discrepancy indices to examine the effect of lemborexant on sleep-wake state discrepancy from baseline through 12 weeks of treatment in patients with insomnia disorder. Relevant confounders were included in the models: age, sex, 3%ODI, BDI-II, and baseline sleep-wake state discrepancy indices. To analyze whether changes in sleep-wake state discrepancy affect changes in sleep quality and daytime sleepiness, a mixed effects model was used. The model included all types of sleep-wake state discrepancy, time, oTST, and age and sex as covariates. Since this was an exploratory study, no correction was made for multiple comparisons. All tests were two-tailed, and alpha was set at 0.05.

### Ethical considerations

This study was conducted in accordance with the approved protocol by the Nagoya University Clinical Study Review Board (2021–0079) and was registered in the Japanese Registry of Clinical Trials (jRCT s041210024). The study was conducted in compliance with the principles of the Declaration of Helsinki and Ministry of Health, Labour and Welfare regulations. Written, informed consent was obtained from all participants prior to enrollment.

## Results

### Demographic information

Clinical background information is presented in Table 1. The mean SOL was more than 30 minutes, and the mean TST was less than 300 minutes, consistent with findings in chronic insomnia, but suggesting a high degree of individual variation. Baseline sleep-wake state discrepancy also varied widely between individuals, with overestimation of sleep-wake state discrepancy observed for SOL and underestimation for WASO, TST, and SE.

**Table 1:** Summary of demographic information of the participants at baseline

*n* = 29	Mean ± SD
Age (years)	58.5 ± 7.6
Sex (female/male)	14(48.3)/15(51.7)
Objective sleep parameter	
oSOL (min)	33.7 ± 27.4
oWASO (min)	51.4 ± 31.3
oTST (min)	287.7 ± 84.8
oSE (%)	76.4 ± 11.0
Subjective sleep parameter	
sSOL (min)	41.0 ± 38.3
sWASO (min)	37.1 ± 38.7
sTST (min)	257.1 ± 95.3
sSE (%)	75.4 ± 21.6
Sleep-wake state discrepancy	
disSOL (min)	−7.3 ± 34.0
disWASO (min)	14.3 ± 45.0
disTST (min)	34.6 ± 74.2
disSE (%)	1.0 ± 20.4
Questionnaire	
PSQI	8.9 ± 2.3
ESS	12.0 ± 4.0
BDI-II	10.0 ± 7.8
Pulse oximetry	
3%ODI (/h)	9.1 ± 7.8

SD, standard deviation; oSOL, objective sleep onset latency; oWASO, objective wake after sleep onset; oTST, objective total sleep time; oSE, objective sleep efficiency; sSOL, subjective sleep onset latency; sWASO, subjective wake after sleep onset; sTST, subjective total sleep time; sSE, subjective sleep efficiency; disSOL, discrepancy of sleep onset latency; disWASO, discrepancy of wake after sleep onset; disTST, discrepancy of total sleep time; disSE, discrepancy of sleep efficiency; PSQI, Pittsburg Sleep Questionnaire Inventory; ESS, Epworth Sleepiness Scale; BDI-II, Beck Depression Inventory-II; ESS, Epworth Sleepiness Scale; 3%ODI, 3% Oxygen Desaturation Index

### Sleep-wake state discrepancy change

**Fig. 1:**
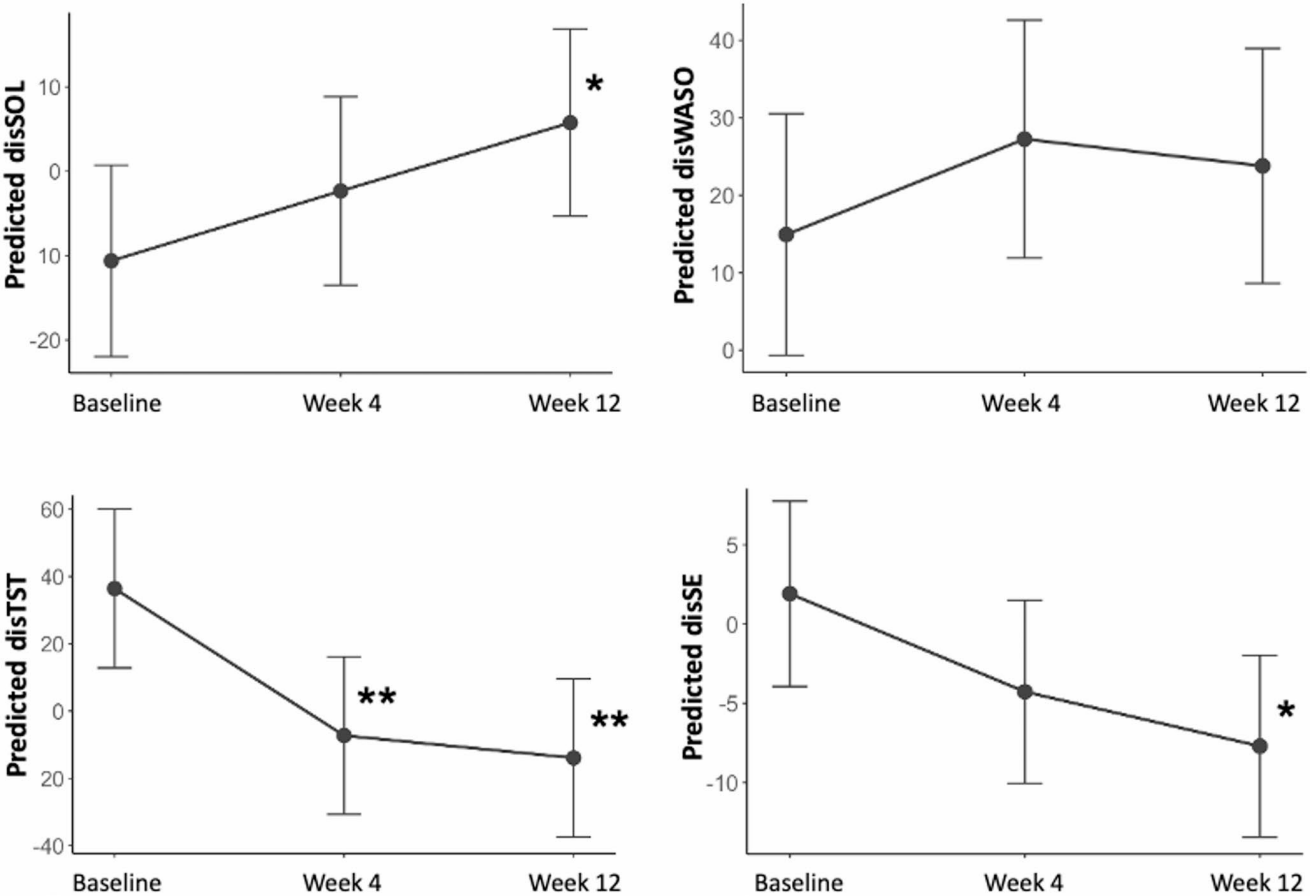
Predicted time series of sleep-wake state discrepancy from baseline to 12 weeks from linear mixed models. Capped vertical lines represent 95% confidence intervals. *p < 0.05, **p < 0.01. disSOL, discrepancy of sleep onset latency; disWASO, discrepancy of wake after sleep onset; disTST, discrepancy of total sleep time; disSE, discrepancy of sleep efficiency

All subjective and objective sleep parameters were improved by lemborexant throughout the study period in the FLUID study, as reported elsewhere (Miyata et al. 2024). The changes in sleep-wake state discrepancies are shown in Fig. 1. The disSOL, disTST, and disSE approached zero over time, indicating that the sleep-wake state discrepancy in these parameters was resolved. Linear mixed effects models of sleep-wake state discrepancy from baseline to week 12 are shown in Table 2. The disSOL increased significantly at week 12 (*p* = 0.035). The disWASO did not change significantly over 12 weeks. The disTST decreased significantly over time (baseline vs. week 4: *p* = 0.004; baseline vs. week 12: *p* = 0.001). The disSE decreased significantly at week 12 (*p* = 0.012).

**Table 2:** Linear mixed effects models of sleep-wake state discrepancy from baseline to 12 weeks

Variable	Estimate	95%CI	t value	*p*-value
disSOL				
Week 4	8.290	−6.313 – 22.893	1.113	0.266
Week 12	16.381	1.148 – 31.614	2.109	**0.035**
Age	0.033	−0.861 – 0.928	0.073	0.942
Sex (female)	5.380	−7.422 – 18.182	0.824	0.410
3%ODI	−0.011	−0.787 – 0.766	−0.027	0.979
BDI-II	0.481	−0.373 – 1.335	1.105	0.269
Baseline disSOL	0.524	0.324 – 0.724	5.151	**< 0.001**
disWASO				
Week 4	12.329	−7.365 – 32.023	1.228	0.220
Week 12	8.863	−11.161 – 28.887	0.868	0.386
Age	−0.034	−1.284 – 1.216	−0.053	0.958
Sex (female)	−4.349	−22.396 – 13.698	−0.473	0.636
3%ODI	0.446	−0.609 – 1.501	0.829	0.407
BDI-II	1.017	−0.196 – 2.229	1.644	0.100
Baseline disWASO	0.404	0.193 – 0.615	3.759	**< 0.001**
disTST				
Week 4	−43.696	−73.268 – −12.124	−2.898	**0.004**
Week 12	−50.344	−80.712 – −19.976	−3.251	**0.001**
Age	−0.391	−2.109 – 1.326	−0.447	0.655
Sex (female)	−0.079	−26.414 – 26.257	−0.006	0.995
3%ODI	−0.835	−2.4565 – 0.785	−1.011	0.312
BDI-II	−1.233	−3.186 – 0.719	−1.238	0.216
Baseline disTST	0.458	0.250 – 0.666	4.317	**< 0.001**
disSE				
Week 4	−6.180	−13.592 – 1.231	−1.635	0.102
Week 12	−9.618	−17.130 – −2.105	−2.510	**0.012**
Age	−0.096	−0.539 – 0.346	−0.426	0.670
Sex (female)	−0.715	−7.157 – 5.728	−0.218	0.828
3%ODI	−0.176	−0.569 – 0.218	−0.876	0.381
BDI-II	−0.374	−0.818 – 0.070	−1.652	0.099
Baseline disSE	0.420	0.259 – 0.581	5.112	**< 0.001**

disSOL, discrepancy of sleep onset latency; disWASO, discrepancy of wake after sleep onset; disTST, discrepancy of total sleep time; disSE, discrepancy of sleep efficiency; 3%ODI, 3% Oxygen Desaturation Index; BDI-II, Beck Depression Inventory-II

### Analysis of the defined sleep-wake state discrepancy group

**Fig. 2:**
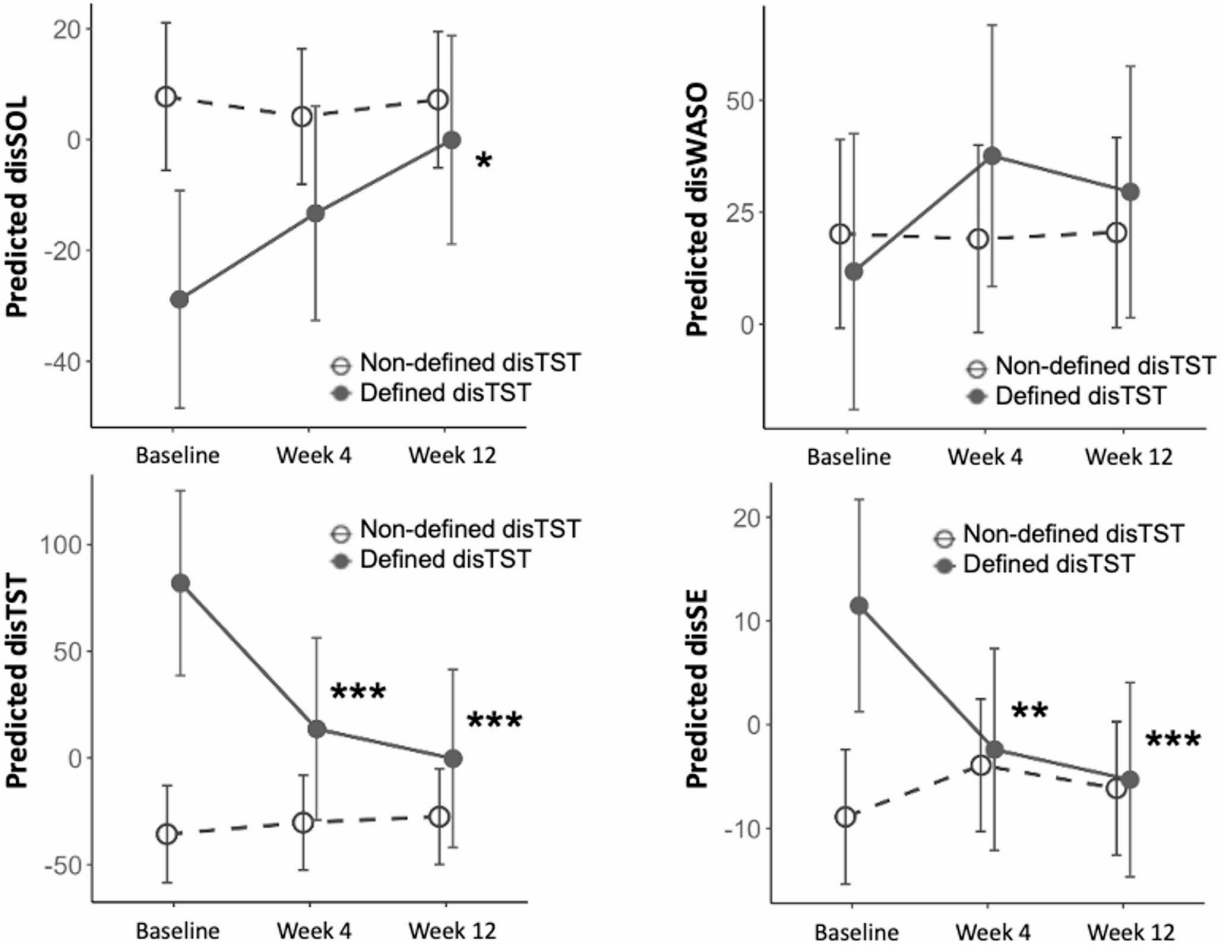
Predicted time series of sleep-wake state discrepancy from baseline to 12 weeks from linear mixed models grouped by defined TST discrepancy. The defined disTST implies oTST-sTST > 0. The non-defined disTST implies oTST-sTST ≤ 0. Capped vertical lines represent 95% confidence intervals. *p < 0.05, **p < 0.01, ***p < 0.001. disSOL, discrepancy of sleep onset latency; disWASO, discrepancy of wake after sleep onset; disTST, discrepancy of total sleep time; disSE, discrepancy of sleep efficiency

A group analysis was performed to determine whether the definition of TST discrepancy (oTST– sTST > 0) was met. The sample size of the sleep-wake state discrepancy definition group was 17 participants, and the sample size of the non-definition group was 12 participants. Linear mixed effects models of sleep-wake state discrepancy from baseline to week 12 by group are shown in Table 3. The disSOL increased significantly in the definition group (baseline vs. week 12: *p* = 0.010), but it did not change significantly in the non-definition group. The disWASO did not change significantly in either group. The disTST and disSE decreased significantly in the definition group (baseline vs. week 4: *p* < 0.001 and *p* = 0.007, respectively; baseline vs. week 12: *p* < 0.001, both), but not significantly in the non-definition group. Sleep-wake state discrepancies except for disWASO approached zero over time. This indicates a reduction in sleep-wake state discrepancy and an improvement in sleep-wake state discrepancy in SOL (disSOL), TST (disTST), and SE (disSE). These changes in the sleep-wake state discrepancy definition group are shown in Fig. 2.

**Table 3:** Linear mixed effects models of sleep-wake state discrepancy from baseline to 12 weeks grouped by defined TST discrepancy

	oTST-sTST > 0 group (*n *= 17)			oTST-sTST ≤ 0 group (*n *= 12)		
Variable	Estimate	95%CI	*p*-value	Estimate	95%CI	*p*-value
disSOL						
Week 4	17.364	-3.494 – 38.221	0.103	-4.282	-22.309 – 13.744	0.641
Week 12	29.659	7.214 – 52.105	**0.010**	0.981	-17.078 – 19.040	0.915
Age	-0.406	-2.053 – 1.242	0.629	0.287	-1.417 – 0.741	0.741
Sex (female)	8.196	-12.658 – 1.242	0.441	9.746	-14.654 – 34.145	0.434
3%ODI	0.193	-1.117 – 1.502	0.773	0.028	-1.028 – 1.084	0.958
BDI-II	1.733	-0.263 – 3.729	0.089	-0.037	-1.115 – 1.040	0.946
Baseline disSOL	0.472	0.121 – 0.822	**0.008**	0.518	0.093 – 0.942	**0.017**
disWASO						
Week 4	21.023	-8.070 – 50.115	0.157	1.258	-20.354 – 22.870	0.909
Week 12	18.620	-11.527 – 48.767	0.226	-0.198	-21.154 – 20.758	0.985
Age	0.094	-2.358 – 2.546	0.940	1.000	-1.678 – 3.678	0.464
Sex (female)	-13.383	-47.146 – 20.381	0.437	7.778	-37.329 – 52.886	0.735
3%ODI	0.424	-1.263 – 2.112	0.622	0.766	-0.904 – 2.435	0.369
BDI-II	1.867	-0.848 – 4.582	0.178	1.297	-0.327 – 2.921	0.117
Baseline disWASO	0.464	0.116 – 0.812	**0.009**	0.564	0.106 – 1.234	0.099
disTST						
Week 4	-74.891	-113.431 – -36.350	**<0.001**	0.684	-29.932 – 31.301	0.965
Week 12	-92.580	-134.513 – -50.648	**<0.001**	6.311	-23.747 – 36.369	0.681
Age	-1.203	-4.393 – 1.987	0.460	0.862	-1.206 – 2.931	0.414
Sex (female)	8.029	-38.254 – 54.313	0.734	3.869	-34.761 – 42.499	0.844
3%ODI	-0.970	-3.964 – 2.023	0.525	-0.575	-2.156 – 1.006	0.476
BDI-II	-2.891	-7.324 – 1.542	0.201	-0.208	-1.915 – 1.498	0.811
Baseline disTST	0.505	0.025 – 0.985	**0.039**	0.285	-0.172 – 0.743	0.221
disSE						
Week 4	-13.974	-24.090 – -3.858	**0.007**	4.715	-3.262 – 12.692	0.247
Week 12	-18.762	-29.228 – -8.297	**<0.001**	2.019	-5.998 – 10.035	0.622
Age	0.060	-0.785 – 0.904	0.890	-0.265	-0.957 – 0.427	0.453
Sex (female)	-0.187	-10.766 – 10.392	0.972	-9.259	-21.922 – 3.405	0.152
3%ODI	-0.187	-0.791 – 0.417	0.544	-0.491	-1.033 – 0.051	0.076
BDI-II	-0.815	-1.783 – 0.154	0.099	-0.220	-0.750 – 0.311	0.417
Baseline disSE	0.381	0.104 – 0.658	**0.007**	0.903	0.431 – 1.375	**<0.001**

disSOL, discrepancy of sleep onset latency; disWASO, discrepancy of wake after sleep onset; disTST, discrepancy of total sleep time; disSE, discrepancy of sleep efficiency; 3%ODI, 3% oxygen desaturation index; BDI-II, Beck Depression Inventory-II

### Clinical significance of changes in sleep-wake state discrepancy

PSQI and ESS were improved by lemborexant throughout the study period in the FLUID study, as reported elsewhere (Miyata et al. 2024). The effect of changes in sleep-wake state discrepancy on changes in PSQI and ESS by the mixed effects model is shown in Table 4. No significant effects of any sleep-wake state discrepancy and time on PSQI change were found, except for oTST (*p* = 0.015). For changes in ESS that represent decreases (improvement), time (week 4: *p* = 0.003; week 12: *p* < 0.001), disTST (*p* = 0.008), and sex (*p* = 0.034) had significant effects, whereas other sleep-wake state discrepancy variables and oTST had no significant effects.

**Table 4:** Mixed effect models for the effect of sleep-wake state discrepancy on PSQI and ESS from baseline to 12 weeks

	PSQI			ESS		
Variable	Estimate	95%CI	*p*-value	Estimate	95%CI	*p*-value
disSOL	0.0003	−0.030 – 0.031	0.982	0.002	−0.047 – 0.051	0.943
disWASO	0.023	−0.004 – 0.050	0.088	0.028	−0.011 – 0.067	0.164
disTST	−0.0003	−0.012 – 0.011	0.954	0.023	0.006 – 0.041	**0.008**
disSE	0.047	−0.050 – 0.143	0.342	−0.020	−0.162 – 0.122	0.780
4 weeks	0.0001	−1.093 – 1.093	1.000	−2.469	−4.112 – −0.825	**0.003**
12 weeks	−0.513	−1.598 – 0.573	0.354	−2.957	−4.604 – −1.310	**< 0.001**
Age	0.036	−0.050 – 0.122	0.416	0.003	−0.132 – 0.137	0.970
Sex (female)	−0.112	−1.351 – 1.127	0.859	−2.147	−4.134 – −0.159	**0.034**
oTST	−0.011	−0.020 – −0.002	**0.015**	−0.012	−0.025 – 0.001	0.082

PSQI, Pittsburg Sleep Questionnaire Inventory; ESS, Epworth Sleepiness Scale; disSOL, discrepancy of sleep onset latency; disWASO, discrepancy of wake after sleep onset; disTST, discrepancy of total sleep time; disSE, discrepancy of sleep efficiency; oTST, objective total sleep time; CI, confidence interval

## Discussion

The present study demonstrated significant improvements in SOL, TST, and SE sleep-wake state discrepancy in individuals with insomnia disorder following 12 weeks of lemborexant treatment. In participants meeting the defined criteria for sleep-wake state discrepancy, these measures showed greater decreases. Importantly, lemborexant did not induce sleep-wake state discrepancy in the group without pre-existing sleep-wake state discrepancy. Furthermore, improvement in TST sleep-wake state discrepancy was associated with reduced daytime sleepiness. These findings suggest that lemborexant effectively alleviates sleep-wake state discrepancy in participants with insomnia disorder in their home environment, and that the observed improvement in daytime sleepiness could be mediated by enhanced sleep perception accuracy. To the best of our knowledge, this is the first study to demonstrate that a hypnotic, specifically lemborexant, improves sleep-wake state discrepancy over an extended period, and that this improvement is associated with improved daytime functioning.

Evidence demonstrating hypnotic-induced improvement in sleep-wake state discrepancy remains limited (Rezaie et al. 2018). Single-dose studies of zopiclone have shown potential improvement in SOL and TST discrepancy through non-rapid eye movement (NREM) sleep stabilization and arousal suppression (Hermans et al. 2021). Though not directly addressing sleep-wake state discrepancy, single-dose triazolam has been shown to blur sleep-wake distinctions following forced awakenings, potentially affecting subjective sleep quality perception (Mendelson 1993). In contrast, single-dose flurazepam showed no significant effect on sleep or wake time after forced awakenings, though it improved arousal thresholds and subjective-objective sleep quality, without affecting time perception (Mendelson et al. 1988). These benzodiazepine receptor agonist (BZRA) studies have notable limitations, including single-dose administration, laboratory rather than natural settings, and indirect sleep-wake state discrepancy assessments. The present study addressed these limitations by prospectively evaluating orexin receptor antagonist effects on sleep-wake state discrepancy in participants’ natural overnight sleep environments at baseline, week 4, and week 12. Of the various sleep-wake state discrepancy measures, the effect on WASO discrepancy was minimal. Though WASO discrepancy has not been extensively studied, evidence suggests that individuals with prolonged WASO may represent a distinct insomnia subtype (Miller et al. 2016). The influence of depression and obstructive sleep apnea has also been noted (Kawai et al. 2022). Future research might explore potential synergistic effects when combining non-pharmacological therapy with careful patient assessment.

As an orexin receptor antagonist, lemborexant improves both subjective and objective sleep measures (Rosenberg et al. 2019), maintains natural sleep architecture (Kron et al. 2024; Moline et al. 2021), and enhances sleep quality (Karppa et al. 2020; Miyata et al. 2024). The meta-analysis also demonstrates lemborexant’s efficacy in both subjective and objective measures during the one-month treatment period, as well as its safety profile, as indicated by low risk of treatment discontinuation (Kishi et al. 2020, 2021). Research has extensively documented the impact of arousal during sleep onset on time perception errors and subjective sleep onset sensations (Hermans et al. 2020a, b). Increased arousal frequency has been shown to affect subjective sleep duration perception (Castelnovo et al. 2021). Neurophysiological features of sleep-wake state discrepancy include cortical hyperarousal during NREM sleep, altered sensory and cognitive processing (Krystal et al. 2002; Perlis et al. 1997), and increased high-frequency EEG activity during both REM and NREM sleep (Lecci et al. 2020). Since lemborexant increases both REM and NREM sleep with the increase of REM stage percentage (Moline et al. 2021), it may be particularly effective in managing sleep-wake state discrepancy. The BZRAs stabilize NREM sleep, reduce arousals, and improve subjective sleep quality (Hermans et al. 2021; Mendelson 1993; Mendelson et al. 1988), since they reduce cortical arousal while simultaneously increasing EEG activity (Stephan and Siclari 2023). Though CBT-I has been well documented to improve SOL and TST discrepancy in individuals with insomnia disorder, predominantly by improving subjective sleep (Dzierzewski et al. 2019; Kay et al. 2015; Lund et al. 2013; Perrault et al. 2022; Spina et al. 2024), many CBT-I studies rely solely on actigraphy, leaving the influence on sleep architecture uncertain. Given these limitations in existing treatments, lemborexant’s demonstrated ability to improve both subjective and objective sleep measures while maintaining normal sleep architecture suggests it may be a promising therapeutic option for sleep-wake state discrepancy.

The treatment of sleep-wake state discrepancy has significant clinical implications. In the present study, improvements in sleep-wake state discrepancy were associated with reduced ESS scores. The ESS, which evaluates daytime sleepiness, is also associated with daytime functioning and quality of life (Fabbri et al. 2021). Though the ESS is frequently used clinically for these evaluations and narcolepsy, studies of daytime sleepiness in insomnia are limited, focusing primarily on conditions such as Parkinson’s disease (Iranzo et al. 2024), circadian rhythm disorders (Sun and Chen 2022), and antiepileptic drug effects (Liguori et al. 2021). Lemborexant has been shown to improve ESS scores as early as week 4, with sustained improvement through 12 weeks (Miyata et al. 2024). Since insomnia disorder diagnosis considers both nocturnal symptoms and daytime functioning, the present study provides novel evidence that improvements in sleep-wake state discrepancy are associated with enhanced daytime functioning. Improvement of sleep-wake state discrepancy may be associated with enhanced quality of life (Cho et al. 2022), improved functioning (Semler and Harvey 2005), and potential prevention of depression (Jackowska et al. 2011) and chronic insomnia (Harvey and Tang 2012; Perlis et al. 1997). These findings underscore the importance of evaluating both objective and subjective dimensions of sleep for comprehensive sleep-wake state discrepancy management (Benz et al. 2023), and in clinical practice, careful use of portable devices that can objectively measure sleep is expected (Kawai et al. 2023; Miyata et al. 2021).

Several limitations of this study should be noted. First, the study design included only a lemborexant treatment group, without active comparator drugs or placebo controls, making it impossible to determine whether the observed effects are specific to lemborexant. Second, limited data analysis, a single-day and small sample size, limited the ability to conduct more sophisticated statistical analyses, such as structural equation modeling. Given the previously demonstrated dose-dependent effects of lemborexant on both subjective and objective outcomes (Kishi et al. 2020, 2021), it is conceivable that its impact on sleep-wake state discrepancy may also vary by dose. However, due to the high collinearity between time and dose in time-series data, as well as the limited sample size, it was not feasible to perform a dose-dependent analysis in the present study. Third, participants were allowed to maintain their typical sleep schedules, unlike standard clinical trials with fixed bedtimes and wake times. Though this introduces potential behavioral variability that may affect results, it enhances the study’s ecological validity by evaluating objective sleep measures in a home environment, providing insights into sleep-wake state discrepancy under real-world conditions. Furthermore, though objective sleep was measured using a portable EEG device, which has demonstrated correlation with PSG (Kawai et al. 2023; Miyata et al. 2021), the results should be interpreted with consideration of the methodological differences between these assessment tools. The evaluation of sleep-wake state discrepancy also varies between laboratory-based PSG and home-based actigraphy, warranting careful interpretation of the findings (Maltezos et al. 2024).

## Conclusion

Lemborexant demonstrated long-term efficacy in improving sleep-wake state discrepancy in participants with chronic insomnia. Notably, its effectiveness was more pronounced in individuals with greater baseline sleep-wake state discrepancy based on TST, and there was no evidence that lemborexant exacerbated this condition. Furthermore, lemborexant not only alleviated nocturnal insomnia symptoms, but also improved daytime sleepiness associated with chronic insomnia. The improvement in daytime function is associated with amelioration of sleep-wake state discrepancy. These findings underscore the potential of lemborexant as a comprehensive treatment for insomnia, addressing both nighttime and daytime impairments.

## Data Availability

The data that support the findings of this study are available from the corresponding author upon reasonable request.

## References

[CR1] Beck AT, Steer RA, Ball R, Ranieri W (1996) Comparison of Beck depression inventories -IA and -II in psychiatric outpatients. J Pers Assess 67:588–597 8991972 10.1207/s15327752jpa6703_13

[CR2] Benz F, Riemann D, Domschke K, Spiegelhalder K, Johann AF, Marshall NS, Feige B (2023) How many hours do you sleep? A comparison of subjective and objective sleep duration measures in a sample of insomnia patients and good sleepers. J Sleep Res 32:e13802 36529876 10.1111/jsr.13802

[CR3] Buysse DJ, Reynolds CF 3rd, Monk TH, Berman SR, Kupfer DJ (1989) The Pittsburgh sleep quality index: a new instrument for psychiatric practice and research. Psychiatry Res 28:193–213 10.1016/0165-1781(89)90047-4 2748771

[CR4] Castelnovo A, Ferri R, Punjabi NM, Castronovo V, Garbazza C, Zucconi M, Ferini-Strambi L, Manconi M (2019) The paradox of paradoxical insomnia: a theoretical review towards a unifying evidence-based definition. Sleep Med Rev 44:70–82 30731262 10.1016/j.smrv.2018.12.007

[CR5] Castelnovo A, Ferri R, Galbiati A, Rossi A, Zucconi M, Castronovo V, Strambi LF, Manconi M (2021) Extreme sleep state misperception: from psychopathology to objective-subjective sleep measures. Int J Psychophysiol 167:77–85 34216692 10.1016/j.ijpsycho.2021.06.011

[CR6] Cho SE, Kang JM, Ko KP, Lim WJ, Redline S, Winkelman JW, Kang SG (2022) Association between subjective-objective discrepancy of sleeping time and health-related quality of life: a community-based polysomnographic study. Psychosom Med 84:505–512 35321997 10.1097/PSY.0000000000001070 PMC9064942

[CR7] Dzierzewski JM, Martin JL, Fung CH, Song Y, Fiorentino L, Jouldjian S, Rodriguez JC, Mitchell M, Josephson K, Alessi CA (2019) CBT for late-life insomnia and the accuracy of sleep and wake perceptions: results from a randomized-controlled trial. J Sleep Res 28:e12809 30609099 10.1111/jsr.12809 PMC6609514

[CR8] Edinger JD, Arnedt JT, Bertisch SM, Carney CE, Harrington JJ, Lichstein KL, Sateia MJ, Troxel WM, Zhou ES, Kazmi U, Heald JL, Martin JL (2021) Behavioral and psychological treatments for chronic insomnia disorder in adults: an American academy of sleep medicine systematic review, meta-analysis, and GRADE assessment. J Clin Sleep Med 17:263–298 33164741 10.5664/jcsm.8988 PMC7853211

[CR9] Fabbri M, Beracci A, Martoni M, Meneo D, Tonetti L, Natale V (2021) Measuring subjective sleep quality: a review. Int J Environ Res Public Health 18:1082 10.3390/ijerph18031082 PMC7908437 33530453

[CR10] Harvey AG, Tang NK (2012) (Mis)perception of sleep in insomnia: a puzzle and a resolution. Psychol Bull 138:77–101 21967449 10.1037/a0025730 PMC3277880

[CR11] Hermans LWA, Nano MM, Leufkens TR, van Gilst MM, Overeem S (2020a) Sleep onset (mis)perception in relation to sleep fragmentation, time estimation and pre-sleep arousal. Sleep Med X 2:100014 33870171 10.1016/j.sleepx.2020.100014 PMC8041114

[CR12] Hermans LWA, van Gilst MM, Regis M, van den Heuvel LCE, Langen H, van Mierlo P, Krijn R, Hoondert B, Maass H, van Dijk JP, Leufkens TRM, Overeem S (2020b) Modeling sleep onset misperception in insomnia. Sleep 43:zsaa014 10.1093/sleep/zsaa014 32016410

[CR13] Hermans LWA, Regis M, Fonseca P, Overeem S, Leufkens TRM, Vermeeren A, van Gilst MM (2021) Assessing sleep-wake survival dynamics in relation to sleep quality in a placebo-controlled pharmacological intervention study with people with insomnia and healthy controls. Psychopharmacology 238:83–94 32939597 10.1007/s00213-020-05660-3 PMC7794103

[CR14] Iranzo A, Cochen De Cock V, Fantini ML, Perez-Carbonell L, Trotti LM (2024) Sleep and sleep disorders in people with parkinson’s disease. Lancet Neurol 23:925–937 38942041 10.1016/S1474-4422(24)00170-4

[CR15] Jackowska M, Dockray S, Hendrickx H, Steptoe A (2011) Psychosocial factors and sleep efficiency: discrepancies between subjective and objective evaluations of sleep. Psychosom Med 73:810–816 22021463 10.1097/PSY.0b013e3182359e77

[CR16] Johns MW (1991) A new method for measuring daytime sleepiness: the Epworth sleepiness scale. Sleep 14:540–545 1798888 10.1093/sleep/14.6.540

[CR17] Karppa M, Yardley J, Pinner K, Filippov G, Zammit G, Moline M, Perdomo C, Inoue Y, Ishikawa K, Kubota N (2020) Long-term efficacy and tolerability of lemborexant compared with placebo in adults with insomnia disorder: results from the phase 3 randomized clinical trial SUNRISE 2. Sleep 43 10.1093/sleep/zsaa123 PMC7487867 32585700

[CR18] Kawai K, Iwamoto K, Miyata S, Okada I, Ando M, Fujishiro H, Noda A, Ozaki N (2022) A study of factors causing sleep state misperception in patients with depression. Nat Sci Sleep 14:1273–1283 35873712 10.2147/NSS.S366774 PMC9296877

[CR19] Kawai K, Iwamoto K, Miyata S, Okada I, Fujishiro H, Noda A, Nakagome K, Ozaki N, Ikeda M (2023) Comparison of polysomnography, single-channel electroencephalogram, fitbit, and sleep logs in patients with psychiatric disorders: cross-sectional study. J Med Internet Res 25:e51336 38090797 10.2196/51336 PMC10753421

[CR20] Kay DB, Buysse DJ, Germain A, Hall M, Monk TH (2015) Subjective-objective sleep discrepancy among older adults: associations with insomnia diagnosis and insomnia treatment. J Sleep Res 24:32–39 25219802 10.1111/jsr.12220 PMC4747029

[CR21] Kishi T, Nomura I, Matsuda Y, Sakuma K, Okuya M, Ikuta T, Iwata N (2020) Lemborexant vs suvorexant for insomnia: a systematic review and network meta-analysis. J Psychiatr Res 128:68–74 32531478 10.1016/j.jpsychires.2020.05.025

[CR22] Kishi T, Nishida M, Koebis M, Taninaga T, Muramoto K, Kubota N, Moline M, Sakuma K, Okuya M, Nomura I, Iwata N (2021) Evidence-based insomnia treatment strategy using novel orexin antagonists: a review. Neuropsychopharmacol Rep 41:450–458 34553844 10.1002/npr2.12205 PMC8698673

[CR23] Kron JOJ, Keenan RJ, Hoyer D, Jacobson LH (2024) Orexin receptor antagonism: normalizing sleep architecture in old age and disease. Annu Rev Pharmacol Toxicol 64:359–386 37708433 10.1146/annurev-pharmtox-040323-031929

[CR24] Krystal AD, Edinger JD, Wohlgemuth WK, Marsh GR (2002) NREM sleep EEG frequency spectral correlates of sleep complaints in primary insomnia subtypes. Sleep 25:630–640 12224842

[CR25] Lecci S, Cataldi J, Betta M, Bernardi G, Heinzer R, Siclari F (2020) Electroencephalographic changes associated with subjective under- and overestimation of sleep duration. Sleep 43:zsaa094 10.1093/sleep/zsaa094 32409833

[CR26] Liguori C, Toledo M, Kothare S (2021) Effects of anti-seizure medications on sleep architecture and daytime sleepiness in patients with epilepsy: a literature review. Sleep Med Rev 60:101559 34710770 10.1016/j.smrv.2021.101559

[CR27] Lund HG, Rybarczyk BD, Perrin PB, Leszczyszyn D, Stepanski E (2013) The discrepancy between subjective and objective measures of sleep in older adults receiving CBT for comorbid insomnia. J Clin Psychol 69:1108–1120 23280680 10.1002/jclp.21938

[CR28] Maltezos A, Perrault AA, Walsh NA, Phillips EM, Gong K, Tarelli L, Smith D, Cross NE, Pomares FB, Gouin JP, Dang-Vu TT (2024) Methodological approach to sleep state misperception in insomnia disorder: comparison between multiple nights of actigraphy recordings and a single night of polysomnography recording. Sleep Med 115:21–29 38325157 10.1016/j.sleep.2024.01.027

[CR29] Mendelson WB (1993) Pharmacologic alteration of the perception of being awake or asleep. Sleep 16:641–646 8290858

[CR30] Mendelson WB, Martin JV, Stephens H, Giesen H, James SP (1988) Effects of flurazepam on sleep, arousal threshold, and the perception of being asleep. Psychopharmacology 95:258–262 3137607 10.1007/BF00174520

[CR31] Miller CB, Bartlett DJ, Mullins AE, Dodds KL, Gordon CJ, Kyle SD, Kim JW, D’Rozario AL, Lee RS, Comas M, Marshall NS, Yee BJ, Espie CA, Grunstein RR (2016) Clusters of insomnia disorder: an exploratory cluster analysis of objective sleep parameters reveals differences in neurocognitive functioning, quantitative EEG, and heart rate variability. Sleep 39:1993–2004 27568796 10.5665/sleep.6230 PMC5070753

[CR32] Miyata S, Iwamoto K, Banno M, Eguchi J, Kaneko S, Noda A, Ozaki N (2021) Performance of an ambulatory electroencephalogram sleep monitor in patients with psychiatric disorders. J Sleep Res 30:e13273 33372341 10.1111/jsr.13273

[CR33] Miyata S, Iwamoto K, Okada I, Fujimoto A, Kogo Y, Mori D, Amano M, Matsuyama N, Nishida K, Ando M, Taoka T, Naganawa S, Ozaki N (2024) Assessing the real-world, long-term impact of lemborexant on sleep quality in a home-based clinical study. Nat Sci Sleep 16:291–303 38524766 10.2147/NSS.S448871 PMC10960545

[CR34] Moline M, Zammit G, Cheng JY, Perdomo C, Kumar D, Mayleben D (2021) Comparison of the effect of lemborexant with placebo and zolpidem tartrate extended release on sleep architecture in older adults with insomnia disorder. J Clin Sleep Med 17:1167–1174 33590823 10.5664/jcsm.9150 PMC8314653

[CR35] Morin CM, Buysse DJ (2024) Management of insomnia. N Engl J Med 391:247–258 39018534 10.1056/NEJMcp2305655

[CR36] Okada I, Iwamoto K, Miyata S, Fujimoto A, Tanaka M, Amano M, Matsuyama N, Taoka T, Naganawa S, Ozaki N (2021) FLUID study: study protocol for an open-label, single-centre pilot study to investigate the effect of lemborexant on sleep management in Japanese sUbjects aged 50 years and older with insomnia disorder. BMJ Open 11:e054885 34836909 10.1136/bmjopen-2021-054885 PMC8727681

[CR37] Perlis ML, Giles DE, Mendelson WB, Bootzin RR, Wyatt JK (1997) Psychophysiological insomnia: the behavioural model and a neurocognitive perspective. J Sleep Res 6:179–188 9358396 10.1046/j.1365-2869.1997.00045.x

[CR38] Perrault AA, Pomares FB, Smith D, Cross NE, Gong K, Maltezos A, McCarthy M, Madigan E, Tarelli L, McGrath JJ, Savard J, Schwartz S, Gouin JP, Dang-Vu TT (2022) Effects of cognitive behavioral therapy for insomnia on subjective and objective measures of sleep and cognition. Sleep Med 97:13–26 35691208 10.1016/j.sleep.2022.05.010

[CR39] Rezaie L, Fobian AD, McCall WV, Khazaie H (2018) Paradoxical insomnia and subjective-objective sleep discrepancy: a review. Sleep Med Rev 40:196–202 29402512 10.1016/j.smrv.2018.01.002

[CR40] Riemann D, Espie CA, Altena E, Arnardottir ES, Baglioni C, Bassetti CLA, Bastien C, Berzina N, Bjorvatn B, Dikeos D, Dolenc Groselj L, Ellis JG, Garcia-Borreguero D, Geoffroy PA, Gjerstad M, Goncalves M, Hertenstein E, Hoedlmoser K, Hion T, Holzinger B, Janku K, Jansson-Frojmark M, Jarnefelt H, Jernelov S, Jennum PJ, Khachatryan S, Krone L, Kyle SD, Lancee J, Leger D, Lupusor A, Marques DR, Nissen C, Palagini L, Paunio T, Perogamvros L, Pevernagie D, Schabus M, Shochat T, Szentkiralyi A, Van Someren E, van Straten A, Wichniak A, Verbraecken J, Spiegelhalder K (2023) The European insomnia guideline: an update on the diagnosis and treatment of insomnia 2023. J Sleep Res 32:e14035 38016484 10.1111/jsr.14035

[CR41] Rosenberg R, Murphy P, Zammit G, Mayleben D, Kumar D, Dhadda S, Filippov G, LoPresti A, Moline M (2019) Comparison of lemborexant with placebo and Zolpidem tartrate extended release for the treatment of older adults with insomnia disorder: A phase 3 randomized clinical trial. JAMA Netw Open 2:e1918254 31880796 10.1001/jamanetworkopen.2019.18254 PMC6991236

[CR42] Semler CN, Harvey AG (2005) Misperception of sleep can adversely affect daytime functioning in insomnia. Behav Res Ther 43:843–856 15896282 10.1016/j.brat.2004.06.016

[CR43] Spina MA, Bei B, Rajaratnam SW, Krystal A, Edinger JD, Buysse DJ, Thase M, Manber R (2024) Cognitive behavioural therapy for insomnia reduces actigraphy and diary measured sleep discrepancy for individuals with comorbid insomnia and major depressive disorder: a report from the TRIAD study. Sleep Med 114:137–144 38183804 10.1016/j.sleep.2023.12.014

[CR44] Stephan AM, Siclari F (2023) Reconsidering sleep perception in insomnia: from misperception to mismeasurement. J Sleep Res 32:e14028 37678561 10.1111/jsr.14028

[CR45] Sun SY, Chen GH (2022) Treatment of circadian rhythm sleep-wake disorders. Curr Neuropharmacol 20:1022–1034 34493186 10.2174/1570159X19666210907122933 PMC9886819

[CR46] Wang Y, Loparo KA, Kelly MR, Kaplan RF (2015) Evaluation of an automated single-channel sleep staging algorithm. Nat Sci Sleep 7:101–111 26425109 10.2147/NSS.S77888 PMC4583116

[CR47] Wenigmann M, Gorzka RJ, Garling M, Spiegelhalder K, Höllmer H, Schulz H (2019) Sleep state misperception in psychiatric patients. Somnologie 23:43–48

